# The ABC of Vitamin D: A Qualitative Study of the Knowledge and Attitudes Regarding Vitamin D Deficiency amongst Selected Population Groups

**DOI:** 10.3390/nu5030915

**Published:** 2013-03-15

**Authors:** Billie Bonevski, Jamie Bryant, Sylvie Lambert, Irena Brozek, Vanessa Rock

**Affiliations:** 1 Priority Research Centre for Translational Neuroscience and Mental Health, Level 5 McAuley Building, Mater Hospital, Cnr Edith & Platt Streets, Waratah, NSW, 2298, Australia; 2 Priority Research Centre in Health Behaviour, School of Medicine and Public Health, University of Newcastle, Hunter Medical Research Institute, Newcastle, 2305, Australia; E-Mail: Jamie.Bryant@newcastle.edu.au; 3 Translational Cancer Research Unit, Ingham Institute for Applied Medical Research, South Western Sydney Clinical School, UNSW Medicine, The University of New South Wales, Sydney, 2170, Australia; E-Mail: s.lambert@unsw.edu.au; 4 Cancer Council NSW, PO Box 572 Kings Cross, NSW, 1340, Australia; E-Mails: irenab@nswcc.org.au (I.B.); vanessar@nswcc.org.au (V.R.)

**Keywords:** vitamin D, focus groups, knowledge and attitudes

## Abstract

Objective: In Australia, vitamin D supply in food is limited, and sun exposure is the main source of vitamin D. However skin cancer risk is high, and the need to gain some sun exposure for adequate vitamin D is challenging public health messages to use protection in the sun. The complex vitamin D public health message may be confusing the public and, in particular, those at highest risk for vitamin D deficiency. This study explored vitamin D and sun exposure attitudes, knowledge and practices of some groups considered at risk of vitamin D deficiency and those delivering healthy sun exposure messages to children. Method: 52 adults participated in six focus groups. Results: Results corroborated with previous research showing low levels of vitamin D knowledge. Individual and environmental barriers to receiving adequate sun exposure were also identified. Conclusions and Implications: The message advocating balanced sun exposure to produce adequate vitamin D needs to be made clearer and be more effectively communicated. Findings provide insights to aid development of appropriate public health messages for safe sun exposure and vitamin D, especially for vulnerable groups.

## 1. Introduction

While there continues to be uncertainty about recent claims of vitamin D’s preventive role with some types of cancers [1,2], autoimmune disorders (such as multiple sclerosis) and possibly cardiovascular diseases [2] and diabetes [3], its essential benefits in the normal development and maintenance of bone health [2,4] have been long known. 

In Australia, vitamin D content in food is almost non-existent, and it is predominantly gained from exposure to ultraviolet (UV) sun exposure. Because of its relationship to UV exposure, vitamin D status is associated with geography. There is greater insufficiency of vitamin D at high latitudes, like northern European countries [5,6,7], but it is also found at low latitudes, like Australia, where UV levels are generally high and rates of skin cancer are amongst the highest in the world [8,9]. This co-occurrence of vitamin D insufficiency and skin cancer is puzzling and has led to the development of guidelines for a balanced approach to sun exposure for both the public [10,11] and health professionals [12]. The balance message suggests some sunlight exposure each day for adequate vitamin D production, but not so much that would lead to increased skin cancer risk.

Population groups at higher risk of vitamin D deficiency include the elderly living in residential care (22%–86% [13,14]), dark skinned and veiled pregnant women (80% [15]), individuals with hip fracture (63% deficiency [16]) and those who cover their skin for religious reasons [17].

Findings from Australian surveys suggest that there is a limited awareness and understanding about vitamin D in the general community [18]. For example, 80% of participants in a community survey were unable to name a health benefit of adequate vitamin D and 15% were unable to give an estimate of the amount of sun exposure needed for vitamin D maintenance [18].

Successful communication of a health message has been associated with changes in people’s beliefs about and attitudes toward a risky behaviour and, in turn, changes in that behaviour [19] and the need for messages to be consistent has been highlighted [20,21]. The current vitamin D and sun “balance” message, however, is complex requiring an up-to-date understanding of factors, such as personal skin type, amount of sun exposure, time of day and UV rating, season, latitude and clothing worn [10,11,12]. While broad community surveys provide valuable prevalence data on the attitudes, knowledge and actions of populations, they often fail to capture more subtle influential factors. For example, is uncertainty regarding amount of sun exposure required for adequate vitamin D [18], due to the sometimes contradictory vitamin D and sun “balance” messages or other factors? Qualitative methods can address these types of issues in the development of public health messages providing a deeper understanding behind reasons for low knowledge or misperceptions. Specifically, focus groups provide an understanding of a target group’s motivations, environments, belief systems and health practices [22,23,24]. To date, no qualitative research exploring these constructs with people with increased risk of vitamin D deficiency or individuals responsible for delivering healthy sun exposure messages to children (teachers) has been published. This study aimed to explore vitamin D and sun exposure attitudes, knowledge and practices of the selected populations using focus groups. 

## 2. Methods

### 2.1. Study Design

Qualitative focus groups (*n* = 6) were conducted in November 2010, in Sydney, Australia. The Consolidated Criteria for Reporting Qualitative Research framework was used to guide the reporting of the findings [25].

Two health behaviour theories—*Social Cognitive Theory* [26] and *The Health Belief Model* [27]—informed sampling, development of the interview guide and analysis. Social Cognitive Theory suggests that behaviour is influenced by social and physical environments, along with the features of the behaviour [26]. The Health Belief Model specifies that individuals adopt a health protective behaviour (e.g., sun protection for skin cancer or sun exposure for vitamin D), to the extent that they perceive themselves to be susceptible to a health threat (*i.e.*, skin cancer or deficiency), perceive the threat to be severe, perceive the benefits of the proposed health action for mitigating the threat and can overcome perceived barriers to the health behaviour. 

### 2.2. Sample & Recruitment

Participants were English-speaking adults aged over 18 years, living in Sydney, Australia. Purposive sampling ensured participants were recruited from the three groups of interest: (1) teachers (two groups—primary and secondary); (2) office workers (two groups); and (3) elderly (two groups—community dwelling and those in residential aged care facilities).

Recruitment of participants was conducted by an accredited recruitment agency—Stable Research. Participants were recruited from pre-existing registers and supplemented by additional methods (e.g., contacting local aged care facilities). Equal numbers of males and females were targeted across groups. Four focus groups were held in a location with a high migrant population to increase the cultural mix of the sample. “Office workers” were defined as those working in an indoor office at least four days a week. “Community living elderly” were persons aged 65 years living independently and “Elderly living in aged care” were persons aged 65 years and over living in an aged care facility or assisted retirement village. 

### 2.3. Procedure

An independent social market research organisation (IPSOS-Eureka) with experience in qualitative research was engaged to conduct the focus groups. Groups were conducted in four locations across Sydney. Following initial telephone contact by the recruitment agency to determine interest in participating, each participant was mailed an information statement and consent form prior to attending the group discussions. All focus groups were recorded. One of the authors was an observer in the focus groups (IB). All participants were offered $80 reimbursement for time and travel expenses. Ethics approval was granted by the University of Newcastle Human Ethics Research Committee.

### 2.4. Discussion Guide Content

Each focus group was led by an experienced moderator who used a discussion guide to focus the discussion. Informed by theoretical models outlined above, the discussion guide included items within the broad domains of: knowledge of vitamin D; awareness of vitamin D message; barriers to receiving adequate sun exposure; and communicating the vitamin D message. 

### 2.5. Data Coding and Analysis

Audio recordings were transcribed verbatim. Transcripts were coded by two independent coders (JB & SL) using NVivo version 8 [28]. Each transcript was reviewed line-by-line, and through inductive reasoning, words, statements and paragraphs related to the broad domains of the interview guide were extracted. Through this in-depth analysis, similar excerpts were identified using the same label or code [29]. Codes were either single words (e.g., “*food*”, “*sun*”) or short phrases (e.g., “*balance between sun exposure and protection*”) that captured the essence of the excerpts. Codes were grouped under broad domains of the discussion guide and theoretical constructs (e.g., personal susceptibility to health effects of vitamin D deficiency). Where appropriate, sub-categories were developed to further describe the categories. Three of the six focus groups were analysed by two coders (JB and SL), with discrepancies in coding discussed until a kappa of >0.6 was achieved across 75% of central nodes. The remaining transcripts (*n* = 3) were coded independently by one coder (JB or SL). 

## 3. Results

Fifty-two participants (23 males, 29 females) took part in six focus groups. Groups contained seven to nine participants and ranged from 1 to 1.5 h in duration. Whilst we aimed to include approximately equal numbers of males and females in each group, the group with primary school teachers included only one male participant (see Table 1).

**Table 1:** Focus group schedule.

	Participants	Male N	Female N
**Group 1**	Office workers	4	5
**Group 2**	Independent living adults (65 years+)	5	4
**Group 3**	Office workers	4	5
**Group 4**	Community aged home residents (65 years+)	4	5
**Group 5**	Primary School teachers	1	6
**Group 6**	Secondary School teachers	5	4

### 3.1. Knowledge

#### 3.1.1. General Vitamin D Knowledge

Most participants felt they knew less about the benefits or role of Vitamin D in comparison to other vitamins. This was mainly attributed to comparatively limited media attention given to vitamin D compared to other vitamins, such as vitamin C or B. Many participants presumed vitamin D had to be essential and offer some health benefits, but few could name what these were. 

#### 3.1.2. Sources of Information about Vitamin D

Several participants could not recall a specific source of information for their knowledge of vitamin D. Sources of information on vitamin D mentioned by participants included (in descending order):

- *Media:* articles in newspapers, magazines and current affairs programs;
- *Doctors:* some participants (primarily with a deficiency in vitamin D) had learnt about vitamin D from their doctor, although often information they had received was limited;
- *Family members/friends:* few participants mentioned that they had heard about vitamin D through family members, friends or significant others;
- *School and further education:* a small number of participants said they had learnt what they know from school or further education.

#### 3.1.3. Vitamin D Testing and Education

Most participants did not know whether their vitamin D level had ever been tested or assumed it had been tested as part of a blood test for a range of things.

“*… I have a cholesterol test usually at least once a year, but I have got no idea whether there is a vitamin D component in that.*” (Independent aged)

Most participants tested and found to be low in vitamin D did not recall being told by their doctor why it was important, the consequences of inadequate vitamin D or how much sun was needed each day to ensure adequate vitamin D. Rather, it was common for participants to report that they had simply been told they “*need to get out in the sun more*” or advised to take a supplement.

“*No, he [doctor] didn’t [explain why Vitamin D was important], he just recommended [...] I take Caltrac with vitamin D. That was it.*” (Office worker)

### 3.2. Awareness

#### 3.2.1. Knowledge of Times and Seasons for Sun Protection

Many participants identified mid-day or the hottest time of the day as the critical period when sun protection is needed. 

Early morning (e.g., before 10:00) or late afternoon (e.g., after 15:00) was perceived as the ideal time to spend time in the sun, because “*you still get the sunshine, but you are not getting it as intense.*” (Primary school teacher).

#### 3.2.2. Knowledge about Amount of Sun Exposure Required for Adequate Vitamin D

Many participants admitted they were unsure of how long was needed to be in the sun for adequate vitamin D. There was a tendency however to overestimate the time required in summer, with 15–20 min being the most common response. Participants identified that time needed in the sun to get enough vitamin D might vary according to age, skin colour/type and nutrition. Discussion about the amount of sun exposure raised many questions regarding the impact of clothing and sunscreen.

“*How much exposure, too, I mean, I was out in the sun yesterday with arms exposed. I mean, is that the same as, do I need 20 min of that, whereas I can stay outside in the nude for three minutes?*” (Primary school teacher)

#### 3.2.3. Knowledge about Groups at Higher Risk of Vitamin D Deficiency

Participants identified groups that may be at increased risk of vitamin D deficiency in Australia, including; the elderly or individuals who might have difficulty getting outside (e.g., immobile due to disability), workers confined to an office during the day, shift workers and those who cover their skin for religious reasons.

### 3.3. Personal Behaviours and Perceived Risk

#### Personal UV Exposure for Adequate Vitamin D

Current guidelines for vitamin D were communicated to participants (see Table 2), and most felt they were getting adequate sunlight in the summer months on most days of the week. Most school teachers and office workers thought they would easily meet the recommendations on most days in summer, spring and autumn, largely through incidental exposure. While most adults over 65 also thought they would meet recommendations on most days, this was often dependent on the weather. Several participants were surprised at the length of exposure needed in winter, and most reported that they would not meet recommendations in winter.

“*No. I don’t think in winter I would in a day. Especially when you have a week, like days of rain and ... I don’t think in winter.*” (Primary school teacher)

**Table 2:** Summary of key Australian guidelines for sun exposure for vitamin D sufficiency for the general population (moderate fair skin) and people at high risk of vitamin D deficiency.

	General Population
a	Fair skinned people can achieve adequate vitamin D levels (>50 nmol/L) in summer by exposing the face, arms and hands or the equivalent area of skin to a few minutes of sunlight on either side of peak UV periods on most days of the week. In winter, in the southern regions of Australia, where UV radiation levels are less intense, maintenance of vitamin D levels may require 2–3 h of sunlight exposure to the face, arms and hands or equivalent area of skin over a week.
b	In Sydney, in December to January (Australian summer), 6 to 8 min at 10 am or 2 pm. In Sydney, in July to August (Australian winter), 26 to 28 min at 10 am or 2 pm or 16 min at 12 noon.
	**People at high risk of vitamin D deficiency**
a	Naturally dark skinned people (Fitzpatrick skin type 5 and 6) are relatively protected from skin cancer by the pigment in their skin; they could safely increase their sun exposure. Other people at high risk of vitamin D deficiency should discuss their vitamin D status with their medical practitioner, as some might benefit from dietary supplementation with vitamin D.
b	Vitamin D supplementation is likely to be required for this population group.

^a^ The Risks and Benefits of Sun Exposure Position Statement. Approved by the Australian and New Zealand Bone and Mineral Society, Osteoporosis Australia, The Australasian College of Dermatologists and the Cancer Council Australia; updated 2007; ^b^ Calcium, Vitamin D and Osteoporosis [12].

Communication of the guidelines prompted questions from participants seeking more detail about the type of exposure needed. Participants were unsure how sunscreen and protective clothing affected the absorption of vitamin D and whether it is possible to “store” vitamin D by having longer period of sun exposure, but on fewer days per week. Several participants also wondered whether it would be equally acceptable to spend a shorter period of time in the more ‘intense’ sunlight during the middle of the day to get a “*boost [...] of vitamin D*” (Independent aged).

### 3.4. Barriers

#### 3.4.1. Barriers to Receiving Sun Exposure for Adequate Vitamin D

A number of barriers to receiving adequate sun exposure were identified: lack of information and knowledge about the effects of vitamin D deficiency, concern about skin cancer and sun burn, ability to go outside, the weather and work. Overall, participants were much more aware of the “SunSmart” message than the “vitamin D” message and almost unanimously more concerned about preventing skin cancer than about ensuring they get enough vitamin D. Two reasons seemed to underpin thesefindings. Promotion of the “SunSmart” message, as well as an awareness of the dangers of skin cancer has led to participants purposefully limiting their sun exposure. In comparison, vitamin D deficiency seemed inconsequential and not serious enough to warrant any specific action.

“*The consequences I think are greater. It’s [skin cancer] deadly, and you die a lot quicker from cancer than you can from vitamin D deficiency*...” (Secondary school teacher)

Several participants highlighted that the “SunSmart” and “vitamin D” messages seem contradictory—on the one hand, the recommendation is to cover up and on the other hand, the recommendation is to expose skin. A few participants also mentioned that in addition to skin cancer, they were concerned about sunburn and, consequently, limited their exposure. Extremes of weather, including the summer heat and wet weather, being unable to go outside due to medical conditions and physical ability were identified as barriers to sun exposure. 

#### 3.4.2. Ways to Address the Barriers to Increase Sun Exposure

The majority of participants’ suggestions to help them meet recommendations for sun exposure centred around increasing incidental exposure, such as by parking the car further away from their destination, a brief walk at lunchtime and eating meals outdoors. Only one office worker and a secondary school teacher identified fortifying food with vitamin D or taking a supplement as a way of receiving adequate vitamin D. 

#### 3.4.3. Communicating the Vitamin D Message

Overall, participants felt the vitamin D message had not been effectively communicated. Participants made recommendations as to how to communicate the vitamin D message (communication medium) and what needs to be communicated (type of message).

*Communication medium:* Television advertising was considered the communication medium of choice, followed by newspapers, magazines and radio. New media, including the internet, Facebook and “pop-up” ads on websites, such as Google, were identified as potentially effective ways of communicating the message, particularly to adolescents and young adults. 

Doctors and pharmacists were considered a good source of information about vitamin D, especially for the elderly. 

*Type of message*: Participants suggested that messages relating to vitamin D should focus on providing education, but stressed that “It’s got to be a simple message. If it’s too complicated, your eyes just glaze over and you think about something else.” (Independent aged). 

While number of participants suggested combining recommendations for acquiring adequate vitamin D with related messages, such as the *Slip! Slop! Slap!* Message, an equal number of participants were concerned this could cause confusion and be counter-productive. There was particular concern that mixed messages could be used as an excuse by children and adolescents to be out in the sun without sun protection. One participant suggested that the message should only be targeted at those who are at-risk. 

## 4. Discussion

This study used qualitative methods to explore understanding and awareness of the vitamin D message and opportunities and barriers to UV exposure. Focus groups were conducted with individuals at risk for vitamin D deficiency, including people who have limited access to outdoor sunlight through the day, including indoor office workers and elderly people in aged care facilities. The study provides new and unique knowledge in this emerging area, with implications for the development of the “balance” message.

### 4.1. Low Knowledge and Awareness about Balancing the Benefits and Risks of Sun Exposure

The almost complete lack of awareness of the balance message and low levels of knowledge about vitamin D were not surprising. Previous quantitative community surveys conducted in Australia, although mostly in Queensland, have found similar results [18,30]. Furthermore, some research suggests that people who intentionally tan claim to do so for their vitamin D status. Thus, the misunderstanding about vitamin D may be placing people at risk of skin cancer. Our participants were unable to name the health benefits of vitamin D with certainty, had little awareness of UV times of the day for adequate vitamin D exposure and most were unaware of the amount of time in the sun they required. Most participants had not had previous exposure to the current guidelines for sun exposure, and when the message was communicated, it prompted several questions. These results and previous quantitative studies suggest that current communication of the “balance” message is not reaching most of the community. Unlike previous studies that have found that the vitamin D message is being misinterpreted, particularly by people who intentionally suntan putting themselves at risk of skin cancer [30], the current study found people continued to heed the sun protection message, even during winter and outside of peak UV times. These results suggest that even those at risk of deficiency are not aware of the need to increase their vitamin D intake. 

One factor, which may be contributing to the low levels of knowledge and difficulty in communicating the “balance” sun exposure message, is the lack of conclusive research evidence regarding how much time the public needs in direct UV exposure in order to assist their vitamin D status. Currently, the message is complex and different according to location, season and individual characteristics. Broad recommendations for the amount of skin an individual needs to expose to the sun and the amount of time to be exposed are based on incomplete data. As further research evidence is gathered, the messages will be made clearer and communicated more confidently. 

### 4.2. High Levels of SunSmart Awareness and Sun Avoidance Behaviours

Slogans, such as *Slip! Slop! Slap!* and SunSmart, have very high public recognition, and there has been considerable policy and practice in place in Australia since the early 1980s that reinforces sun protective behaviour [31]. Two related themes emerged in the discussions that reflect this situation. Firstly, the SunSmart message has been effectively communicated and adopted by study participants. Use of sun protection amongst this group was largely “normalised”, and most participants reported frequently using sun protection measures. Secondly, the message that skin cancer is a high risk concern had been effectively communicated and taken up by participants. Participants stated they were more concerned about skin cancer than vitamin D deficiency. 

One consequence of the success of the SunSmart messages is that some participants reported the need to use sun protection at all times, with some participants feeling that there was no safe time to be exposed, especially in summer. Encouragingly, most participants identified the middle of the day as unsuitable, and some believed that early morning or late afternoon were suitable for safe sun exposure. No participants offered “outside of peak UV times” as appropriate times to be in the sun. Instead, generic “early” and “late” in the day times were offered. This suggests that the UV Alert, which may be an appropriate tool for displaying safe and unsafe sun exposure times of the day, requires further promotion and education for people to understand and use the ratings. 

### 4.3. Barriers to Sun Exposure for Vitamin D

Incidental exposure during the day was the most common type of sun exposure reported, with more time spent outside on weekends than weekdays. This type of sun exposure may not be sufficient for vitamin D, given that the recommendation of up to 10 min outside of peak UV time is based on estimates from ideal conditions of sun exposure (clear, open sky and an unshaded, horizontal surface) and may not correspond to typical outdoor behaviours [32]. This is the first study to explore barriers to sun exposure for vitamin D. Types of barriers participants in all groups reported included lack of knowledge about the need for vitamin D, concerns about sunburn, the need to use sun protection when outdoors and environmental barriers, including the weather (wet, hot and cold weather), work hours indoors (indoor workers group) and physical inabilities to go outside (aged care group). Development of vitamin D public health education and campaigns need to address these barriers.

### 4.4. Strategies to Overcome the Barriers

Increasing incidental sun exposure through routine, daily, outdoor activities was the main strategy identified by participants for increasing sun exposure for vitamin D. Examples included parking the car ten minutes away from work and walking the distance and eating lunch outside the workplace. Improving education about the need for sun exposure and vitamin D was also suggested. Channels for communicating the vitamin D message included the mass media and internet, schools, doctors and pharmacists. While the mass media has been informally used to date, it’s reporting of health news is less than optimal [33] and has been accused of misrepresenting the vitamin D issue and confusing the message further [34,35]. Alternatively, doctors and pharmacists may be better placed as providers of information, where the benefits and risks can be communicated in a balanced manner and various factors considered in calculating risk and need (including latitude, skin type, season and time of day). Doctors, in particular, frequently need to manage the communication of uncertainties, risks and benefits of medical therapies [36]. However, the results of this study suggest that participants’ doctors were not informing their patients about their vitamin D status. Other research suggests this may be due to low levels of knowledge about vitamin D amongst doctors [37]. Clearly, further efforts into educating both the media and health professionals about vitamin D is needed.

There was a division of opinion regarding whether the vitamin D message should be linked to the *Slip! Slop! Slap!* *Seek and Slide* message. Whilst some participants felt it was a natural way to communicate the balance message, others believed this strategy would confuse the messages and cause negative consequences, including increased tanning. Others have suggested that the vitamin D message can complement SunSmart messages in Australia [31], particularly if the UV Alert is effectively incorporated. Some research has started to suggest that younger tanners are using the need for vitamin D as a reason for their sun tanning behaviours [38]. This highlights the need to carefully explore all the factors that may affect the interpretation and use of a balance message among a variety of target groups and identify methods for communicating the message. A tailored approach with messages designed for different groups may be the most effective and safe. The need for a consistent and simple message was reinforced.

### 4.5. Study Strengths and Weaknesses

This study is one of the first qualitative studies of the knowledge and attitudes of groups at risk of vitamin D deficiency. The use of the theoretical models proved instrumental to shaping aspects of the study. The focus groups found that social and physical environments, as proposed in the Social Cognitive Model, played a role in lack of UV exposure, both for office workers and elderly persons. As predicted by the Health Belief Model, perceived susceptibility to skin cancer and sunburn was greater than risk of vitamin D deficiency, and perceptions of the severity of the skin cancer and sunburn threats were greater than the threat of vitamin D deficiency. An examination of the barriers to the desired health behaviour revealed suggestions for overcoming the barriers. As a result, the study provides valuable insight into peoples’ understanding of the vitamin D and sun exposure message. Other strengths of this formative research are its inclusion of groups at higher risk for vitamin D deficiency and a high number of participants. The use of multiple researchers to collect and analyse the qualitative data also reduced the potential for investigator bias in interpreting the findings. The generalizability of study results is limited, due to two main reasons. First, only select groups were included in the study. Secondly, detailed demographic or skin cancer history information about participants was not collected. Further research is required to generalise these findings to other types of community groups or individuals, including those with darker skin types or those who wear veiled clothing. 
